# Supplementary material for: Transcriptome Analysis Reveals Gemykibivirus Infection Induces Mitochondrial DNA Release in HEK293T Cells
Source: Viruses. 2025 Sep 30;17(10):1331. doi: 10.3390/v17101331 (PMC12567786; doi:10.3390/v17101331)
Supplement: Supplementary file 1 [file viruses-17-01331-s001.zip › viruses-3914459-supplementary.pdf]

Supplementary Figures

Supplementary Figure S1

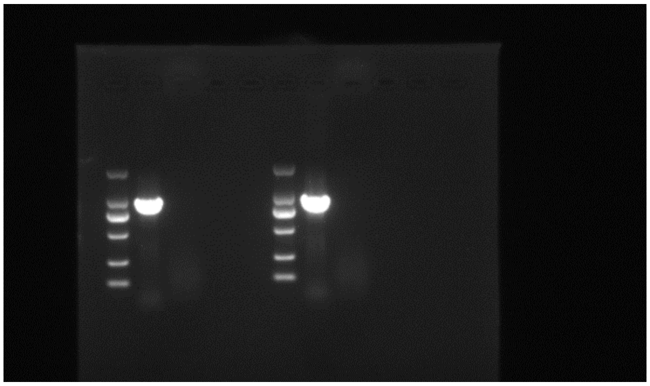

Figure S1. Full electrophoresis image corresponding to Figure 1A. Original scanned image showing detection of the *CAP* gene in the supernatant of HEK293T cells by polymerase chain reaction, visualized using 1% agarose gel electrophoresis.

Supplementary Figure S2

A B

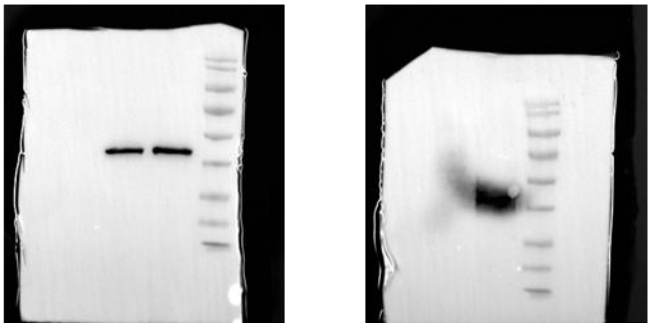

Figure S2. Full blots images corresponding to Figure 1C. Original scanned western blot images showing the expression of Cap protein (A) and β-actin (B) in HEK293T cells during infection.

Supplementary Figure S3

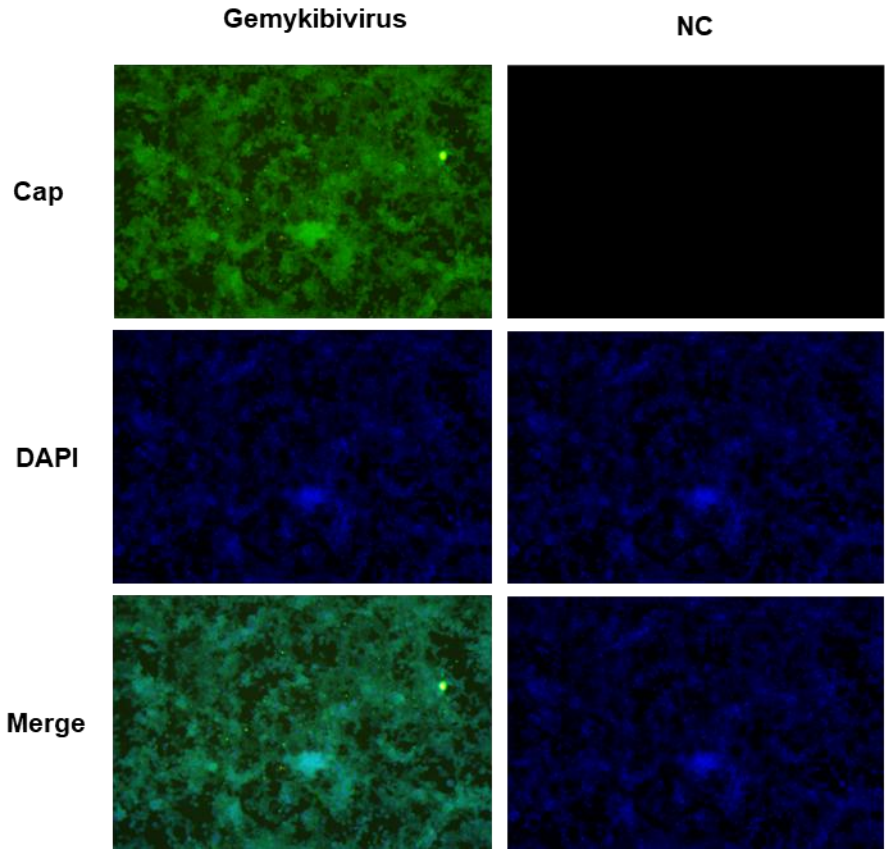

Figure S3. Full confocal microscopy images to Figure 1B.

Supplementary Figure S4

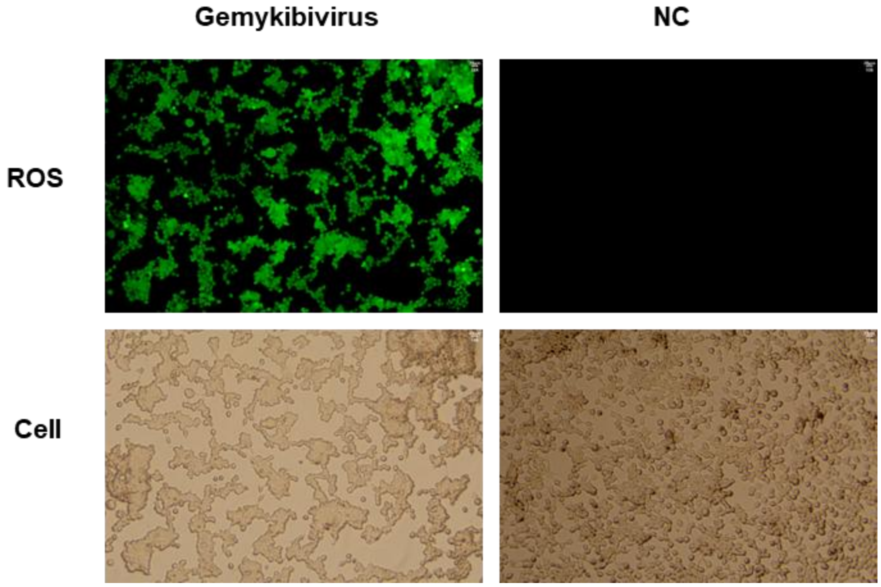

Figure S4. Full confocal microscopy images to Figure 5D.
